# Complete Genome Sequences of Three *Cupriavidus* Strains Isolated from Various Malaysian Environments

**DOI:** 10.1128/genomeA.01498-16

**Published:** 2017-01-19

**Authors:** Nur Asilla Hani Shafie, Nyok-Sean Lau, Hema Ramachandran, Al-Ashraf Abdullah Amirul

**Affiliations:** a Centre for Chemical Biology, Universiti Sains Malaysia, Penang, Malaysia; b School of Biological Sciences, Universiti Sains Malaysia, Penang, Malaysia; c Quest International University, Ipoh, Perak, Malaysia

## Abstract

*Cupriavidus* sp. USMAA1020, USMAA2-4, and USMAHM13 are capable of producing polyhydroxyalkanoate (PHA). This biopolymer is an alternative solution to synthetic plastics, whereby polyhydroxyalkanoate synthase is the key enzyme involved in PHA biosynthesis. Here, we report the complete genomes of three *Cupriavidus* sp. strains: USMAA1020, USMAA2-4, and USMAHM13.

## GENOME ANNOUNCEMENT

*Cupriavidus* spp. are Gram-negative, aerobic, nonsporulating, and rod-shaped bacteria (1). These bacterial strains have great potential in producing polyhydroxyalkanoate (PHA), a class of biologically synthesized plastics with similar physical properties as synthetic plastics (2, 3). The three *Cupriavidus* sp. strains USMAA1020, USMAA2-4, and USMAHM13 were isolated from various natural environments, such as soils and activated sludge, in Malaysia.

The genomic DNA of these strains was extracted using a HiYield genomic DNA minikit (Real Biotech Corporation, Taiwan). The whole-genome sequencing was performed using single-molecule real-time sequencing technology (SMRT) with the PacBio RS II system (Pacific Biosciences, USA). The 10-kb SMRTbell libraries were constructed, and the size selection of the libraries was performed using the Blue Pippin system (Sage Sciences, USA). Each library was sequenced using two SMRT cells with P6-C4 chemistry. *De novo* assembly was carried out using the Hierarchical Genome Assembly Process (HGAP) version 3 (4), and the assemblies were polished using Quiver as included in the SMRT Analysis software.

The genome assemblies of *Cupriavidus* sp. strains USMAA1020, USMAA2-4, and USMAHM13 resulted in three contigs, two circular chromosomes, and one megaplasmid, with a total number of bases, number of reads, and an average read length, respectively, of 1.84 Gb, 159,227, and 11,585 bp for USMAA1020; 2.02 Gb, 143,552 and 14,073 bp for USMAA2-4; 1.87 Gb, 132,876, and 14,078 bp for USMAHM13. The genome sizes are 7,949,449 bp, 8,698,226 bp, and 7,820,821 bp, with G+C contents of 68.5%, 68.0%, and 68.6%, respectively. Genome annotation was performed using Rapid Annotations using Subsystems Technology (RAST) version 2.0 (5, 6). There were 7,133 predicted protein coding sequences (CDSs) and 94 RNA genes in USMAA1020; 7,881 CDSs, and 94 RNA genes in USMAA2-4; and 7,014 CDSs and 95 RNA genes in USMAHM13.

These genomes revealed that *Cupriavidus* sp. strains USMAA1020, USMAA2-4, and USMAHM13 possess polyhydroxyalkanoate synthases (PhaC), which are the key enzymes involved in PHA synthesis with different substrate specificities that determine the monomer composition of the PHA produced.

### Accession number(s).

The complete genome sequences of *Cupriavidus* sp. strains USMAA1020, USMAA2-4, and USMAHM13 have been deposited in GenBank under the accession numbers CP017754 to CP017756 for USMAA1020, CP017748 to CP017750 for USMAA2-4, and CP017751 to CP017753 for USMAHM13.
